# Expression of the Chemokine Receptor CXCR7 in CXCR4-Expressing Human 143B Osteosarcoma Cells Enhances Lung Metastasis of Intratibial Xenografts in SCID Mice

**DOI:** 10.1371/journal.pone.0074045

**Published:** 2013-09-10

**Authors:** Patrick Brennecke, Matthias J. E. Arlt, Roman Muff, Carmen Campanile, Ana Gvozdenovic, Knut Husmann, Nathalie Holzwarth, Elisabetta Cameroni, Felix Ehrensperger, Marcus Thelen, Walter Born, Bruno Fuchs

**Affiliations:** 1 Laboratory for Orthopedic Research, Department of Orthopedics, Balgrist University Hospital, Zürich, Switzerland; 2 Institute of Veterinary Pathology, Division of Immunopathology, University Zurich, Switzerland; 3 Institute for Research in Biomedicine, Bellinzona, Switzerland; University of Florida, United States of America

## Abstract

More effective treatment of metastasizing osteosarcoma with a current mean 5-year survival rate of less than 20% requires more detailed knowledge on mechanisms and key regulatory molecules of the complex metastatic process. CXCR4, the receptor of the chemokine CXCL12, has been reported to promote tumor progression and metastasis in osteosarcoma. CXCR7 is a recently deorphanized CXCL12-scavenging receptor with so far not well-defined functions in tumor biology. The present study focused on a potential malignancy enhancing function of CXCR7 in interaction with CXCR4 in osteosarcoma, which was investigated in an intratibial osteosarcoma model in SCID mice, making use of the human 143B osteosarcoma cell line that spontaneously metastasizes to the lung and expresses endogenous CXCR4. 143B osteosarcoma cells stably expressing *LacZ* (143B-*LacZ* cells) were retrovirally transduced with a gene encoding HA-tagged CXCR7 (143B-*LacZ*-X7-HA cells). 143B-*LacZ*-X7-HA cells co-expressing CXCR7 and CXCR4 exhibited CXCL12 scavenging and enhanced adhesion to IL-1β-activated HUVEC cells compared to 143B-*LacZ* cells expressing CXCR4 alone. SCID mice intratibially injected with 143B-*LacZ*-X7-HA cells had significantly (p<0.05) smaller primary tumors, but significantly (p<0.05) higher numbers of lung metastases than mice injected with 143B-*LacZ* cells. Unexpectedly, 143B-*LacZ*-X7-HA cells, unlike 143B-*LacZ* cells, also metastasized with high incidence to the auriculum cordis. In conclusion, expression of the CXCL12 scavenging receptor CXCR7 in the CXCR4-expressing human 143B osteosarcoma cell line enhances its metastatic activity in intratibial primary tumors in SCID mice that predominantly metastasize to the lung and thereby closely mimic the human disease. These findings point to CXCR7 as a target, complementary to previously proposed CXCR4, for more effective metastasis-suppressive treatment in osteosarcoma.

## Introduction

Osteosarcoma (OS) is the most common primary bone tumor in young adolescents [1]. It occurs with an incidence of approximately 3 cases per million people per year. The survival of OS patients undergoing surgery and radiotherapy alone is poor [2]. Multi-agent chemotherapy increased the 5-year overall survival of patients with localized disease to between 60 and 70% [3]. The survival of patients with metastatic disease, however, remains poor with survival rates ranging from 11 to 20%. Thus, metastasis is the major cause of death in OS [4,5].

Metastasis is a complex multistep process. Tumor cells need to be programmed for local tissue invasion, intravasation, survival in the circulation, migration to and extravasation in secondary organs and finally colonization in the metastatic niche [6]. Tumor cell migration from the primary tumor to secondary organs (tumor cell homing) is frequently guided by chemokines. This has been well documented in breast cancer for the chemokine CXCL12 that, through interaction with its receptor CXCR4 in metastasizing tumor cells, directs their homing to the metastatic site [7,8]. In OS, CXCL12 interacting with CXCR4 was shown to drive tumor progression and metastasis [9,10]. Recently, CXCR7 was deorphanized as a second receptor with high affinity for CXCL12 [11]. CXCR7 was found to be expressed in hematopoietic cells where it functions as scavenger receptor shaping CXCL12 gradients, which in turn enable cell migration mediated by CXCR4 [12]. It was also recognized in many tumor cell lines and in activated endothelial cells [11]. This suggested that CXCR7, like CXCR4, might play a role in immune-regulation, angiogenesis and adhesion to endothelial cells. Interestingly, a number of studies that investigated potential roles of CXCR7 in tumor biology revealed malignancy-enhancing properties of the receptor in different tumor types. Over-expression of CXCR7 in breast cancer cells promoted growth and survival and enhanced adhesion to interleukin-activated HUVEC cells [11,13]. *In vivo*, these breast cancer cells, stably expressing CXCR7, formed larger primary tumors than CXCR7-negative control cells. Furthermore, in an experimental metastasis model of breast cancer, forced expression of CXCR7 enhanced metastatic seeding and proliferation in the lung [14]. These observations were in good agreement with the results of a study investigating patients with breast cancer. This study revealed an inverse correlation between CXCR7 expression in tumor tissue and overall patient survival, suggesting that CXCR7 contributed to a more aggressive tumor phenotype. A tumor and metastasis promoting function of CXCR7 was also described in prostate and lung cancer [14,15]. In rhabdomyosarcoma, shown to employ CXCR4-mediated mechanisms [16,17], ectopic expression of CXCR7 in rhabdomyosarcoma cells enhanced their metastatic potential in experimental metastasis revealed by intravenous tumor cell injection. Furthermore, in Ewing’s sarcoma, up-regulated expression of CXCR4 in tumor tissue specimens was found associated with a high propensity for metastasis and the expression of CXCR7 was an indicator for poor patient survival [16–18]. These findings suggest synergistic malignancy-enhancing actions of CXCR4 and CXCR7 in cancer development. Furthermore, CXCR4-targeting metastasis suppressive treatment strategies with CXCR4 antagonists in experimental metastasis models of OS and in a wide range of other cancer metastasis models were only partially successful [9,19,20].

In view of so far non-existing data on the role of CXCR7 in interaction with the reported tumor and metastasis-promoting actions of the CXCL12/CXCR4 axis in OS [9,10], we investigated in the present study the biological relevance of CXCR7 in a metastasizing intratibial human xenograft OS model in SCID mice that makes use of the human CXCR4-expressing 143B cell line and closely mimics the human disease with metastasis to the lung. The study monitored intratibial primary tumor growth over time and lung metastasis at sacrifice of mice, which were intratibially injected with *LacZ* gene transduced 143B cells that had been superinfected with an empty retroviral vector (143B-LacZ-EV cells) (control) or with the same vector encoding HA-tagged CXCR7 (143B-LacZ-HA-X7 cells). X-gal staining of *LacZ-*expressing 143B cells in tissues dissected from mice at sacrifice allowed the detection of single tumor cells. CXCR7 function in 143B-LacZ-HA-X7 cells was examined in a CXCL12-scavenging assay and *in vitro* metastatic properties by adhesion to IL-1β-stimulated HUVEC.

## Results

### Expression of HA-CXCR7 in 143B-*LacZ* cells mediates CXCL12 scavenging and enhances adhesion to HUVEC.

**Figure 1:**
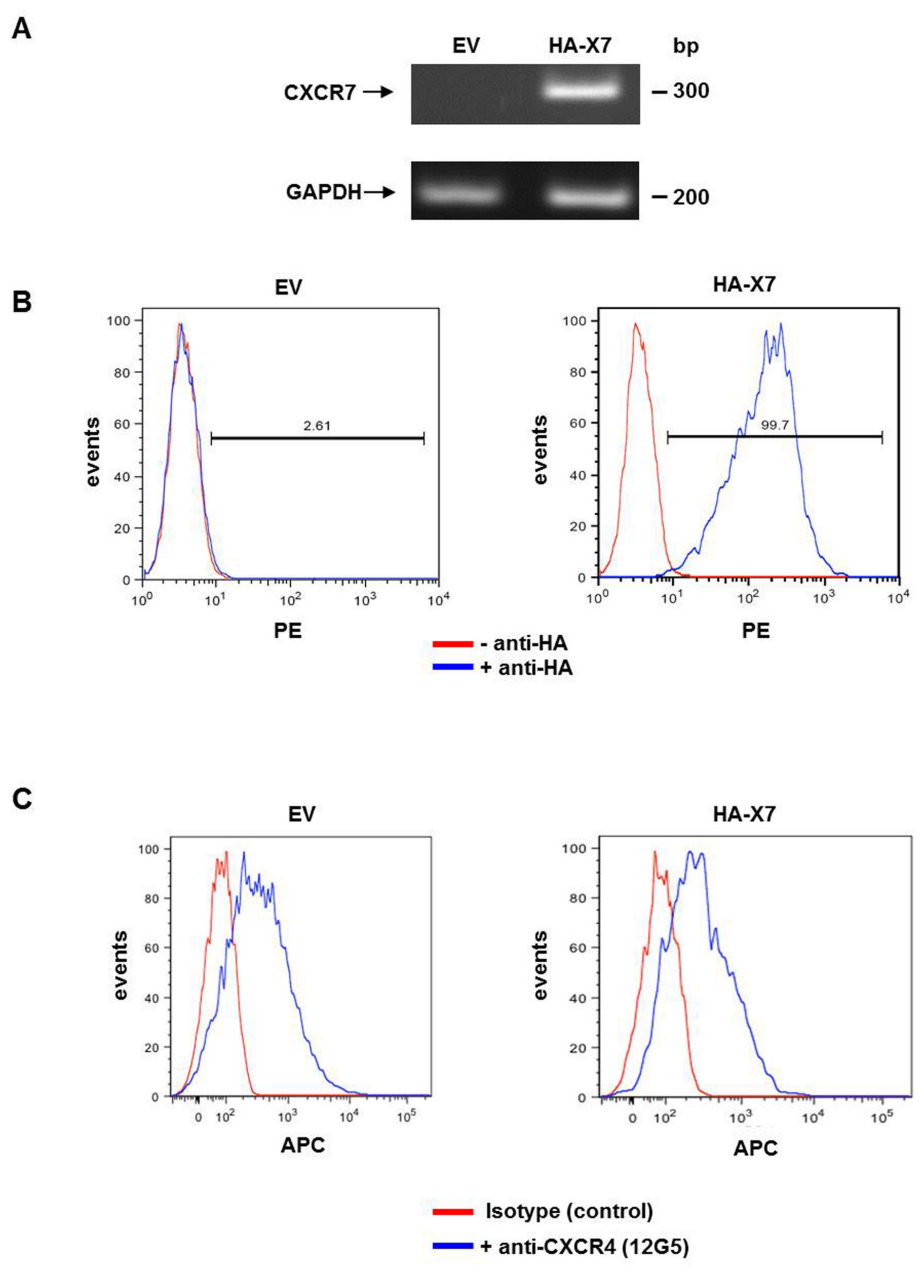
Overexpression of HA-CXCR7 in human OS 143B-*LacZ* cells. (A) Semi-quantitative RT-PCR analysis showed non-detectable expression of CXCR7 in 143B-*LacZ*-EV (EV) cells and abundant HA-CXCR7 (HA-X7) expression in 143B-*LacZ*-HA-X7 cells. GAPDH was used as reference gene. (B) FACS analysis showed HA-CXCR7 cell surface expression in HA-immunostained (blue profiles) 143B-*LacZ*-HA-X7 cells, which was not observed in 143B-*LacZ*-EV (EV) cells. Red profiles were obtained in the absence of HA antibody (n≥3). (C) FACS analysis with 12G5 antibody showed comparable CXCR4 surface expression in 143B-*LacZ*-EV and 143B-*LacZ*-HA-X7 cells (blue profiles), confirming that the overexpression of CXCR7 did not alter the endogenous levels of CXCR4. Red profiles indicate cells that were analyzed in the absence of 12G5 antibody (n=2).

The expression of HA-CXCR7 was verified by semi-quantitative RT-PCR of total RNA extracted from 143B-*LacZ-*EV and 143B-*LacZ-*HA*-*X7 cells (Figure 1A) and by FACS of the cell lines immunostained for HA-CXCR7 cell-surface expression (Figure 1B). Importantly, the expression of CXCR4 in 143B-*LacZ* cells was not affected by the overexpression of HA-CXCR7 as demonstrated by FACS analysis (Figure 1C). The mean CXCR4-related fluorescence intensity of 143B-*LacZ-*HA*-*X7 and of 143B-*LacZ*-EV cells was 859 ± 90 (SEM) and 870 ± 98 (SEM), respectively.

**Figure 2:**
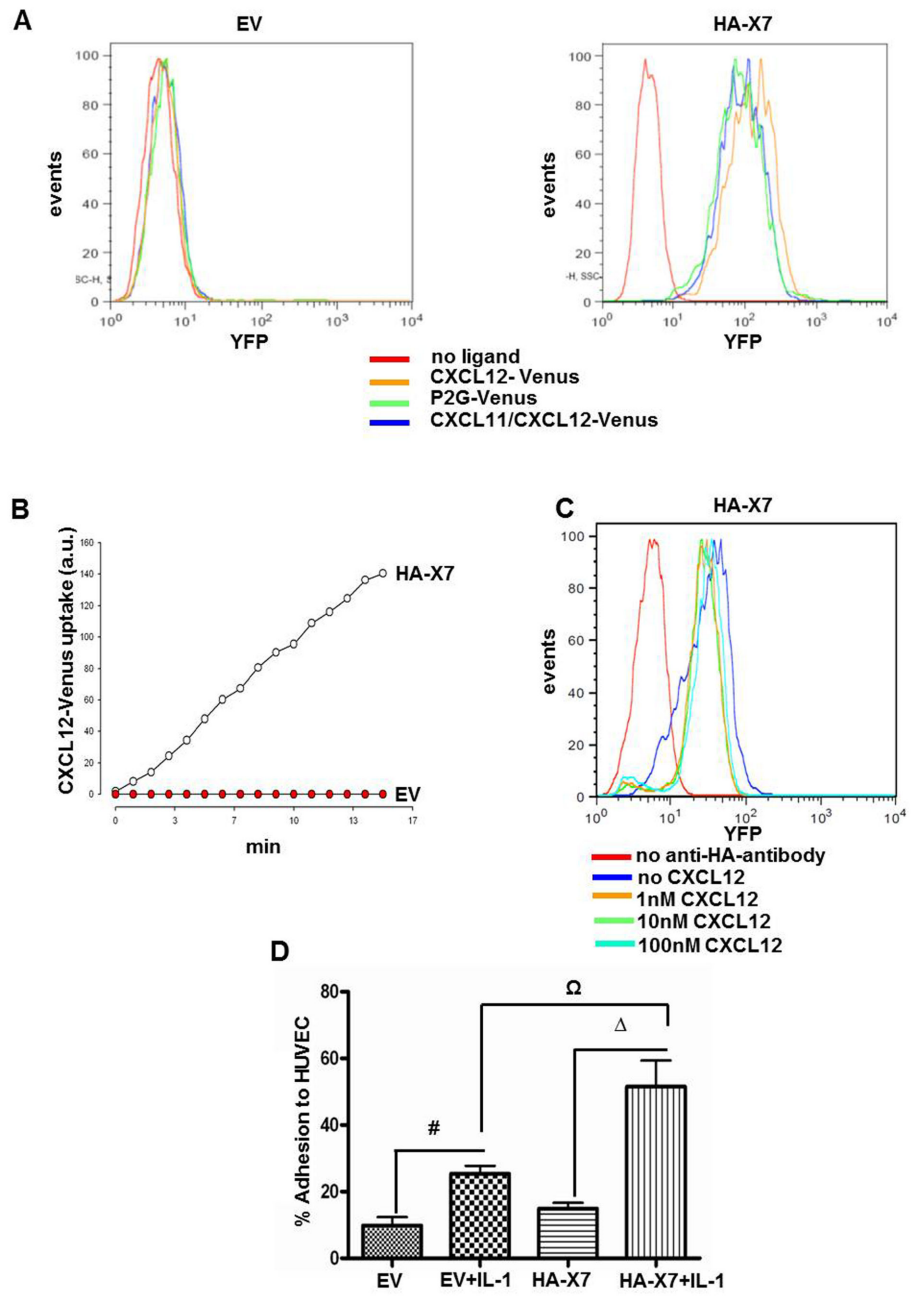
HA-CXCR7 overexpression in 143B OS cells enhanced CXCL12 scavenging and adhesion to HUVEC. (A) FACS analysis showed efficient scavenging of fluorescent Venus-tagged CXCL12 (orange profile), CXCL12 antagonist P2G (green profile) and chimeric CXCL11-CXCL12 (blue profile) in 143B-*LacZ*-HA-X7 (HA-X7), but not in control 143B-*LacZ*-EV (EV) cells. Controls in the absence of fluorescent ligands (red profiles). (B) Time-dependent uptake of CXCL12-Venus by 143B-*LacZ*-HA-X7 (○), assessed by time-laps confocal microscopy was not observed in 143B-*LacZ*-EV (●) cells. (C) CXCL12 at indicated increasing concentrations provoked minimal loss of HA-CXCR7 at the surface of 143B-*LacZ*-HA-X7 analyzed by FACS. (D) 143B-*LacZ*-HA-X7 (HA-X7) cells exhibited enhanced adhesion to interleukin-1β activated HUVEC compared to 143B-LacZ-EV cells. #,Ω,Δ indicate significant differences (p<0.05) between indicated cell lines and conditions. Values represent the mean ± SEM (n=3).

Ligand scavenging by CXCR7 was investigated in 143B-*LacZ-*EV and 143B-*LacZ-*HA*-*X7 cells with three different ligands fused to the fluorescent protein Venus. These ligands included CXCL12-Venus, the competitive CXCR4 antagonist P2G-CXCL12-Venus and chimeric CXCL11/CXCL12-Venus (Figure 1A) [21]. CXCL11 is a second high-affinity ligand of CXCR7 [30]. Time-dependent uptake of the three ligands was only observed in 143B-*LacZ-*HA*-*X7 cells, but not in 143B-*LacZ-*EV cells lacking CXCR7 (Figure 2A, B and movie S1 “CXCL12 -Venus uptake in 143B cells transduced with CXCR7” and movie S2 “CXCL12-Venus uptake transduced with an empty vector construct”, supporting information). Incubation of 143B-*LacZ-*HA*-*X7 cells with increasing concentrations of CXCL12, on the other hand, reduced the density of HA-CXCR7 detected by FACS at the cell surface only minimally (Figure 2C). Taken together, both observations are consistent with reported rapid cycling of HA-CXCR7 between the plasma membrane and endosomal structures [12]. P2G-CXCL12-Venus and CXCL11/CXCL12-Venus were found to be internalized by CXCR7, but it was barely visible in cells that expressed CXCR4 alone. In previous report it was shown [12] that CXCR4 scavenging activity is weak and is probably a consequence of the stoichiometric uptake and degradation with the receptor, whereas CXCR7 can undergo multiple rounds of ligand uptake leading to a marked uptake over time (Figure 2B). Moreover, it has been reported that CXCL12 binds to CXCR7 with a 10x higher affinity (KD~0.2-0.4 nM) than to CXCR4 (KD~2-4 nM) [11,22,23]. Thus, all these parameters together result in CXCR7-mediated ligand internalisation that largely predominates that of CXCR4 under the experimental conditions used in this study.

The effect of CXCR7 overexpression in 143B-*LacZ* cells on adhesion to endothelial cells was studied with non-stimulated and IL-1β-stimulated HUVEC. The adhesion of 143B-*LacZ*- HA*-*X7 cells to non-stimulated HUVEC was the same as that of control 143B-*LacZ-*EV cells (Figure 2D). IL-1β stimulated the adhesion of both cell lines to HUVEC (p<0.05), but, importantly, 143B-*LacZ*-HA-X7 adhered to IL-1β stimulated HUVEC significantly better than 143B-*LacZ*-EV cells (p<0.05).

Taken together, enhanced CXCL12 scavenging and adhesion to HUVEC indicated functional CXCR7 expression in 143B-*LacZ-*HA*-*X7 cells. These findings set the stage for an *in vivo* study that investigated in the 143B-*LacZ* cell line-derived metastasizing intratibial OS model in SCID mice a potential malignancy-enhancing function of CXCR7 in CXCR4 expressing OS.

### CXCR7 overexpression in 143B-LacZ cells diminishes intratibial primary tumor growth, but promotes lung and auriculum cordis metastases in SCID mice

**Figure 3:**
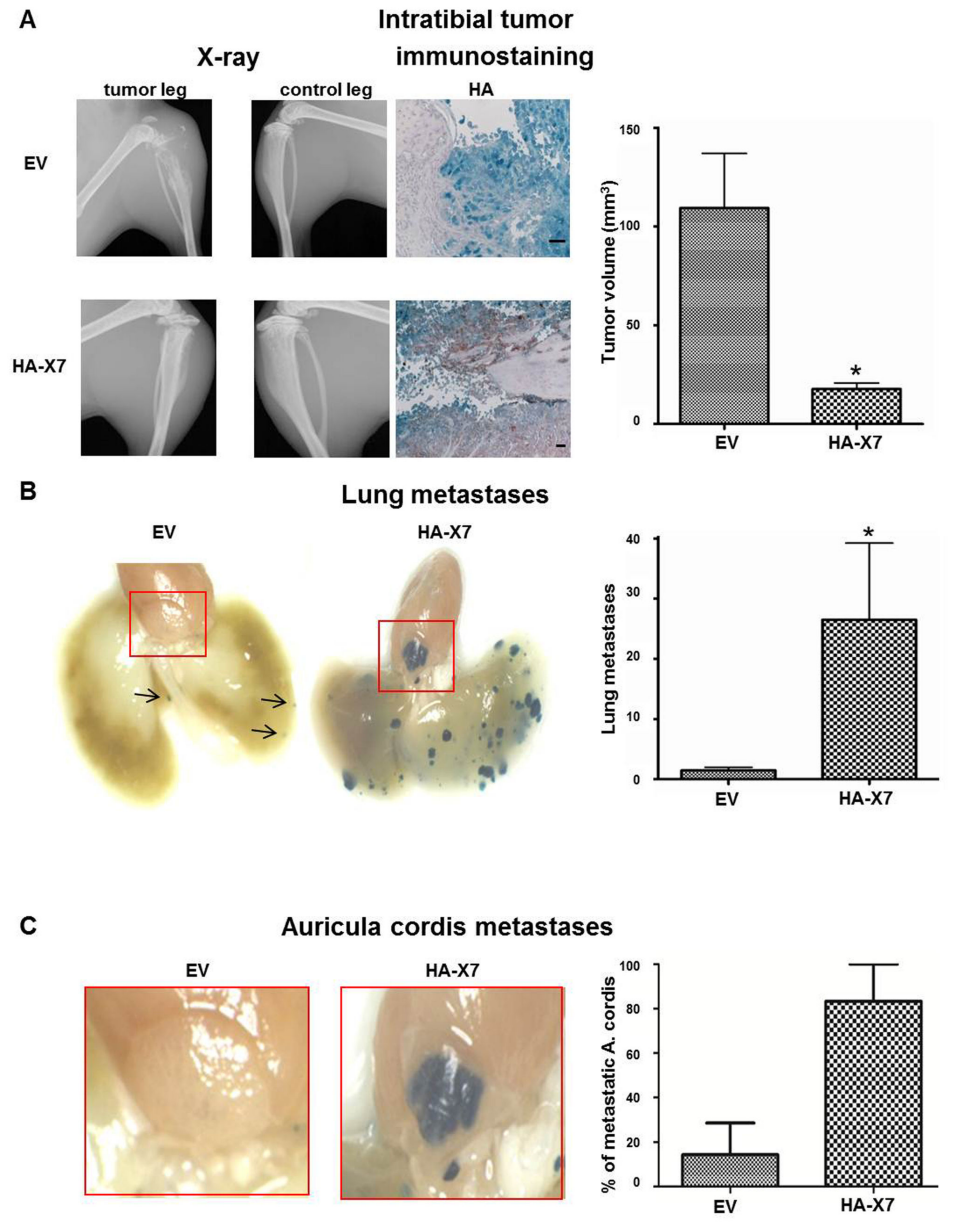
Overexpression of HA-CXCR7 in 143B-*LacZ* cells diminished primary intratibial tumor growth, but promoted metastasis to the lung and the auriculum cordis. (A) Representative X-ray images of a tumor cell injected leg (tumor leg) and of the contralateral non-injected leg (control leg) of mice injected with 143B-*LacZ*-EV (EV) cells or with 143B-*LacZ*-HA-X7 (HA-X7) cells at day 27 after tumor cell injection and representative HA immunohistochemical staining of HA-CXCR7 on sagittal sections of EV and HA-X7 cell injected tibiae and mean calculated volume of intratibial tumors in EV and HA-X7 cell-injected mice at day 27 after tumor cell injection. (B) Representative lung whole mounts with X-gal-stained metastases (blue) obtained from EV (arrows indicate the metastases) and HA-X7 cell injected mice and mean (± SEM) numbers of metastases on the lung surface of respective animals. (C) Whole mounts of auriculum cordis enlarged from (B) with X-gal stained metastatic lesions and mean (± SEM) percentage of mice in indicated groups with auriculum cordis metastatic lesions. Six and 7 mice in the EV and the HA-X7 groups, respectively, were analyzed. *p≤0.05.

Forced expression of HA-CXCR7 in intratibial primary tumors derived from 143B-*LacZ-*HA-X7 cells was confirmed by HA and CXCR7 immunostaining of sagittal paraffin-sections of the tumor-bearing tibia (Figure 3A and Figure S1A). Importantly, the expression pattern of CXCR7 in 143B-*LacZ*-EV and 143B-*LacZ*-HA-X7 cell-derived tumors was indistinguishable from that observed in the respective cells *in vitro*, with non-detectable CXCR7 expression in 143B-*LacZ*-EV cell-derived tumors and robust expression in 143B-*LacZ*-HA-X7 cell-derived tumor tissue (Figure S1A). Moreover, the expression pattern of CXCR4 observed *in vivo* was also comparable to that observed in 143B-*LacZ*-EV and 143B-*LacZ*-HA-X7 cells in vitro, indicating that the in vivo environment did not alter the expression of endogenous CXCR4 in the two cell lines (Figure S1B).

Remarkably, SCID mice injected with 143B-*LacZ-*HA-X7 cells developed within 27 days smaller (p<0.05) intratibial primary tumors than the mice injected with the control 143B *LacZ-*EV cells (Figure 3A and Table 1). However, 143B-*LacZ-*HA-X7 cell-injected mice had significantly (p<0.05) higher numbers of X-gal-stained lung metastases than the control 143B-*LacZ-*EV cell-injected animals (Figure 3B and Table 1). Moreover, rare metastases in the auriculum cordis were observed in only 14% of the 143B-*LacZ-*EV cell-injected SCID mice and surprisingly in 83% of the 143B-*LacZ-*HA-X7 cell-injected animals (Figure 3C and Table 1). This unexpected finding prompted a detailed immunohistochemical analysis of the auriculum cordis and of the lung for expression of endogenous CXCL12 as a chemo-attractant for CXCR4 and even more for CXCR4/CXCR7 co-expressing tumor cells.

**Table 1:** Total number of mice per experimental group and characteristics of primary tumors and metastatic spread.

	143B-*LacZ*-EV		143B-*LacZ*-HA-X7
Number of mice	6		7
Tumour volume (median in mm^3^)	130.5		15.36
Tumour volume (mean ± SEM in mm^3^)	107.2 ± 27.13		17.75 ± 3.08
Number of lung metastatic foci per lung lobe (mean ± SEM)	1.5 ± 0.48		26.67 ± 12.8
Percentage of mice with metastases in the auriculum cordis (mean ± SEM)	14.29 ± 14.29		83.3 ± 16.67

SEM: standard error of the mean

### Immunohistochemistry identified CXCL12 in lung peribronchial tissue and in heart vessels next to the auriculum cordis

**Figure 4:**
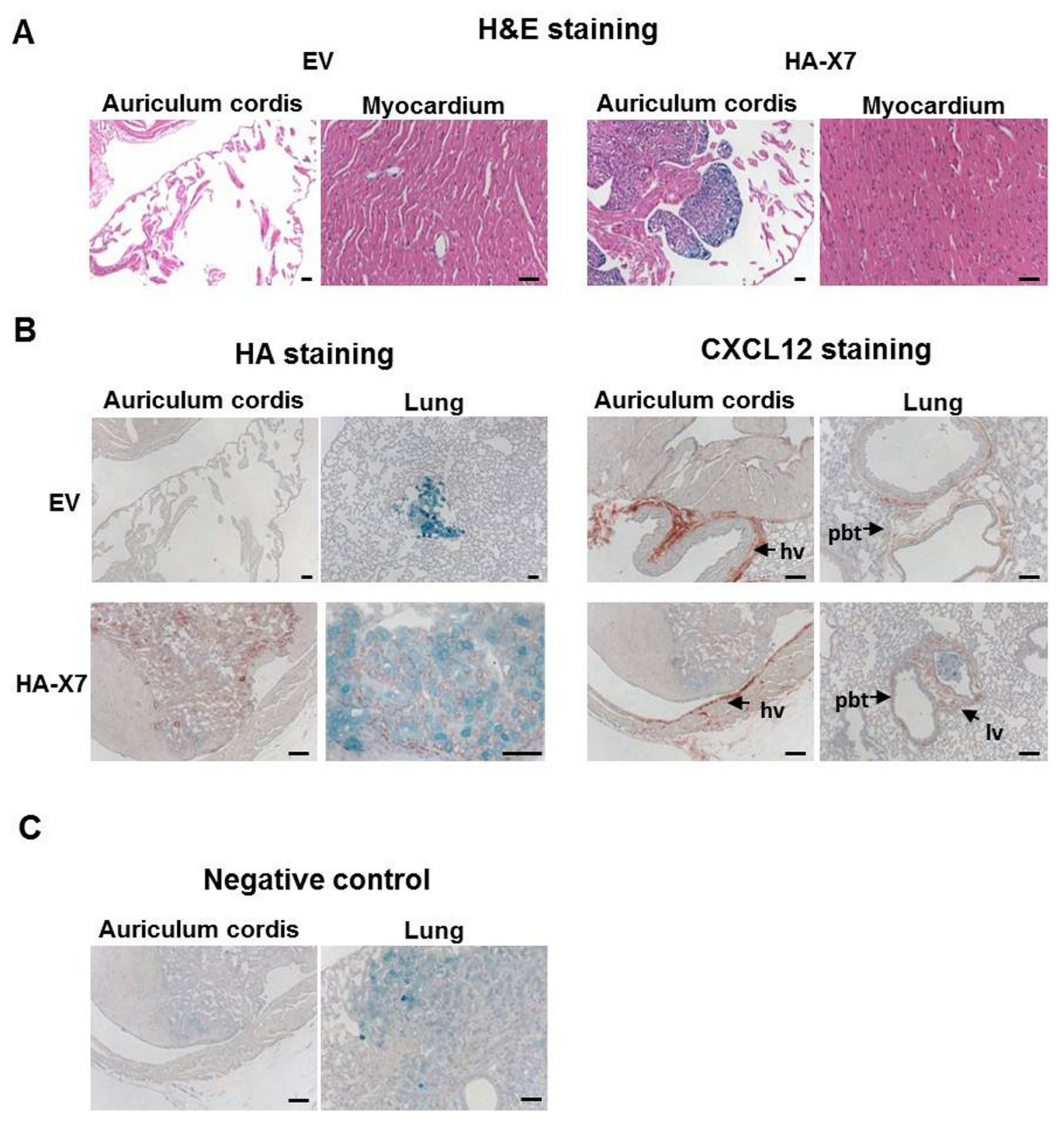
Histology and immunostaining of paraffin sections showed a lower metastatic load in the auriculum cordis and the lung of 143B-*LacZ*- EV than of 143B-*LacZ*-HA-X7 cell-injected mice and expression of CXCL12 in both tissues. (A) Representative H&E staining of sections of indicated tissues collected from 143B-*LacZ*-EV (EV) and 143B-*LacZ*-HA-X7 (HA-X7) cell injected mice. (B) X-gal (blue) and HA immunostaining of metastatic lesions and immunohistochemical localization of CXCL12 in large vessels of the heart base (hv) region and in lung vessels (lv) and in peribronchial tissue (pbt) of representative paraffin sections of indicated tissues collected from EV and HA-X7 cell-injected mice. (C) Control staining of indicated tissues in the absence of primary HA antibodies. Size bars: 50 µm.

Hematoxylin and eosin staining and HA immunostaining of tissue sections of the auriculum cordis confirmed the pronounced infiltration of the organ by 143B-*LacZ*-HA-X7 cells, whereas metastatic lesions remained largely undetectable in the tissue of SCID mice with 143B-*LacZ*- EV cell-derived primary tumors (Figure 4). The combined histological and immunohistochemical analysis confirmed the findings of the X-gal staining of lung and auriculum cordis whole mounts (Figure 3B, C) Interestingly, sections of the myocardia of 143B-*LacZ*-EV cell- and 143B-*LacZ*-HA-X7 cell-injected mice showed no metastatic lesions, suggesting rather selective homing of the cells to the auriculum cordis.

Immunohistochemical staining of lung and heart tissue sections for CXCL12 localized the chemokine in peribronchial tissue and in heart vessels in the proximity of the auriculum cordis (Figure 4B) thereby confirming that lung tissue is a rather rich source of CXCL12. Somewhat surprisingly, CXCL12 remained undetectable in the auriculum cordis of all SCID mice investigated here.

## Discussion

The CXCL12/CXCR4 axis was shown to be involved in tumor development and metastatic spread of many cancer types, including OS [7,9,19,24]. Furthermore, there is emerging evidence for important functions of CXCR7, a recently deorphanized second CXCL12 receptor, in cancer progression and metastasis of different tumor types including those of mesenchymal origin [14–17,25].

The present study focused on the biological relevance of CXCR7 in OS in interaction with the CXCR4/CXCL12 axis, reported to promote primary tumor growth and metastasis [9,10]. Previous studies in other tumor types suggested a malignancy-enhancing activity of CXCR7 even in the absence of CXCR4 [14,15,25]. The results of this study in the intratibial, spontaneously metastasizing OS model in SCID mice confirmed a malignancy enhancing activity of CXCR7 in CXCR4-expressing OS, reflected by more aggressive metastasis to the lung and surprisingly also to the auriculum cordis. Interestingly and surprisingly, forced expression of CXCR7 in the here used intratibial OS xenograft model not only promoted metastasis, but it also diminished primary tumor growth. All these findings suggest that CXCR7 might facilitate extravasation of metastasizing OS cells from the primary tumor site, which is also consistent with the observed remarkable increase in the number of metastases in distant organs. This interpretation of our observation is in good agreement with conclusions of previous studies with CXCR4 and CD34 expressing hematopoietic stem cells (HSC) [26]. These studies showed that CXCL12, produced at high levels in bone marrow, caused retention of HSC in distinct compartments. Administration of the CXCR4 antagonist AMD 3100 reversed this observation and led to a release of HSC progenitor cells from the bone marrow and fast generalized leukocytosis, demonstrating the importance of CXCL12 as a retention signal for HSC in the bone [27–29]. In addition, efficient CXCL12 scavenging by 143B-*LacZ*-HA-X7 cells shown *in vitro* may at the primary tumor site also diminish CXCR4-mediated tumor growth promoting activity of CXCL12 in the human 143B OS cell line. Growth-stimulating activity of CXCL12 has been reported in previous studies in other OS cell lines [9,10]. Contrasting findings of Wang and co-workers who demonstrated that expression of CXCR7 in a CXCR4 background in prostate cancer led to faster primary tumor growth [15,30] suggest that growth regulating functions of CXCL12 may depend on tumor cell origin and on the tumor environment.

The characterization of CXCR7-overexpressing 143B cells *in vitro* revealed increased adhesion to HUVEC and confirmed reported observations in CXCR7 transduced breast cancer cells [11,31]. Based on recently published findings of Zabel and co-workers, who demonstrated that CXCR4/CXCR7 co-expressing lymphoblastic tumor cells exhibited significantly enhanced trans-endothelial migration compared to cells expressing CXCR4 alone [13,31]. We believe that enhanced adhesion of CXCR7 overexpressing 143B cells to lung endothelium leads to more frequent successful trans-endothelial migration and, consequently, to the observed higher number of lung metastatic foci. Thus, the combination of facilitated extravasation of CXCR7 overexpressing 143B cells from the intratibial injection site into the bloodstream, due to enforced local CXCL12 scavenging, and more effective adhesion/TEM at the vasculature/lung interface likely explains the enhanced metastatic potential of CXCR7 overexpressing compared to control 143B cells that resulted in the observed significant increase in the number of lung metastases.

The high incidence of remarkably large metastatic lesions in the auriculum cordis of SCID mice with CXCR7 overexpressing primary tumors compared to 143B control cell-injected animals was an unexpected finding, because, as mentioned before, CXCL12 remained undetectable in this tissue. However, CXCL12 was found expressed in heart vessels in close proximity to the auriculum cordis. Along these lines, the results of a recently reported study showed that CXCR7 enhanced CXCL12/CXCR4-mediated trans-endothelial migration of tumor cells [31]. Moreover, another study demonstrated that CXCL12-evoked chemotaxis of CXCR4 expressing HOS cells to fibronectin was mediated by α4β1 and α5β1 integrins [32] and that intradermal tumors shrunk in response to treatment with α5β1 antibodies. We therefore hypothesize that the here observed metastasis of the HOS cell-derived 143B cells to the auriculum cordis, shown to be enhanced by forced expression of CXCR7, may be also mediated by integrins interacting with extracellular matrix characteristic for this tissue. However, we speculate that the massive metastatic load of CXCR7 overexpressing 143B cells to the auriculum cordis is specific to this OS mouse model because metastasis to this organ has, to our knowledge, so far not been reported for OS patients [33–36].

In conclusion, the results of the present study demonstrate that forced expression of the recently de-orphanized CXCL12-scavenging receptor CXCR7 in the human metastatic CXCR4-expressing 143B OS cell line remarkably enhances its metastatic phenotype *in vitro* and *in vivo*. These findings obtained in an experimental OS model, which closely mimics the human disease, suggest that co-expression of CXCR4 and CXCR7 is also a metastasis-enhancing principle in the human disease that needs to be further investigated. Along these lines, the results of a recent preliminary immunohistochemical analysis of a limited number of human OS tissue specimens, carried out in our laboratory, indicated a tendency for worse survival of patients with tumors that co-expressed CXCR4 and -7 compared to patients with tumors that expressed none of the receptors. However, co-expression of CXCR4 and CXCR7 in OS tumor tissue did not change the predictive power for a tendency of poor outcome that was also already evident when CXCR4 immunostaining was analyzed alone. Nevertheless, this analysis needs to be extended to a larger patient cohort that will allow a meaningful statistical analysis. All observations of the present study taken together suggest that OS treatment that targets both CXCR7 and CXCR4 will likely be more effective than so far reported attempts to inhibit OS progression and metastasis in experimental OS animal models by blocking CXCR4 alone [20].

## Materials and Methods

### Cell lines

143B cells were purchased from the European Collection of Cell Cultures (Salisbury, UK). Cells were grown in Dulbecco’s modified Eagle medium (DMEM) (4.5g/L glucose) and Ham F12 (F12) medium (1:1) supplemented with 2 mM L-glutamine (PAA GmbH, Germany), 10% heat-inactivated fetal calf serum (FCS) (GIBCO, Basel, Switzerland). The cells were kept in a humidified atmosphere of 95% air and 5% CO_2_ at 37°C.

### RNA isolation and RT-PCR

Total RNA was isolated from cell lines with TriReagent (Sigma-Aldrich, St. Louis, MO) as described recently [37]. The RNA was quantified spectrophotometrically and the integrity of the RNA was assessed by standard agarose gel-electrophoresis. The expression of mRNA encoding CXCR7 was analyzed by RT-PCR and GAPDH transcripts were used as a reference. cDNA was synthesized from 1 µg of total RNA with the High Capacity cDNA Reverse Transcription Kit (Applied Biosystems, Zug, Switzerland), using the protocol recommended by the supplier. PCR reactions of 50 µl final volume contained 1X PCR buffer, 0.4 µl of the reverse transcription reaction, 1.25 U Taq polymerase (5Prime), 200 µM of each dNTP, and 0.2 µM of forward and reverse primer pairs specific for the individual transcripts (GAPDH Forward: TGA ACG GGA AGC TCA CTG GCA TGG; GAPDH Reverse: TGG GTG TCG CTG TTG AAG TCA GAG GAG; CXCR7 Forward: 5’ GCA GAG CTC ACA GTT GTT GCA AAG’3; CXCR7 Reverse: 5’ ATG AAG GAG AGC GTG TAG AGC AGG’3) The cDNA was denatured at 94°C for 3 min, and then amplified by 27 to 40 PCR cycles (denaturation at 94°C for 40 sec, primer pair- dependent annealing at 67°C to 69°C for 40 sec, and elongation at 72°C for 20 sec) followed by a final elongation step at 72°C for 7 min. PCR products were analyzed by agarose gel electrophoresis.

### Retroviral transduction of 143B cells

143B cells were transduced with the *LacZ* gene (143B-*LacZ* cells) using the pQCLIN retroviral expression vector (Clontech, Paolo Alto, CA) as recently described for other OS cell lines [38,39]. Subcloning of a HindIII-EcoRV fragment encoding CXCR7 with an N-terminal HA- epitope tag (HA-X7) from a pcDNA3.1 plasmid into the retroviral expression vector pQCIXH containing a hygromycin resistance gene revealed pQCIXH-HA-X7. Retroviral particles with integrated pQCLIN-*LacZ* (*LacZ-*retrovirus), pQCIXH (empty vector (EV)-retrovirus) or pQCIXH-HA-X7 (HA-X7-retrovirus) were produced in HEK293-T cells as reported [38].

143B-*LacZ* cells were infected with EV- (143B-*LacZ*-EV cells) or HA-X7-retrovirus (143B-*LacZ*-X7-HA cells) with the protocol that was used for *LacZ*-transduction of the 143B cells and clones of 143B-*LacZ*-EV and 143B-*LacZ*-X7-HA cells were selected in tissue culture medium containing 1 mg/ml hygromycin (Merck, Zug, Switzerland). In order to further enrich HA-X7 expressing 143B-*LacZ* cells, cells under hygromycin selection were detached with 1xPBS, 0.05% EDTA and resuspended in 1xPBS. They were then stained with 5µg/ml primary HA antibodies (HA 2.11 Covance, Princeton, NJ) at 4°C for 30 min and with 5µg/ml secondary goat anti-mouse IgG-PE (Invitrogen, Carlsbad, CA) at 4°C for 20 min. Subsequently, they were filtered through a filter of 50 µm pore size (Partec, Nunningen, Switzerland) and FACS sorting was performed (FACS Aria, BD Biosciences, San Jose, CA) and analyzed on a FACS machine (Calibur, BD, Franklin Lakes, NJ). 143B-*LacZ*-EV cells as well as 143B-*LacZ*-HA-X7 enriched for HA-CXCR7 expression were further analyzed for comparable expression of CXCR4. Both cell lines were stained with 5µg/ml primary CXCR4 antibody (BD Bioscience, San Jose, CA) directly coupled to the fluorochrome allophycocyanin (APC) at 4°C for 30’ or with an isotype control antibody coupled to APC (BD Bioscience, San Jose, CA) and then also analyzed by FACS.

### CXCR7-mediated ligand internalization examined by time-laps confocal microscopy and by FACS analysis

Venus-fluorescent derivatives of CXCL12 (CXCL12-Venus), its antagonist P2G (P2G-Venus) and of chimeric CXCL12/CXCL11 (CXCL12/CXCL11-Venus) were obtained by fusion with the fluorescent protein Venus and used for ligand internalization experiments. They were produced with a baculovirus expression system in the insect cell line High-5 (Invitrogen, Carlsbad, CA) that was grown in protein-free medium. The fusion proteins were purified by ion-exchange and gel filtration chromatography. The fluorescent ligands were stored in stock solutions at 5 µM concentrations.

Uptake of recombinant CXCL12-Venus by 143B-LacZ-HA-X7 and 143B-LacZ-EV cells was examined by time-laps confocal microscopy as follows: cells were detached with trypsin/EDTA and resuspended in tissue culture medium at a final density of 3-4 x 10^6^ cells/ml. 200 µl of the suspension were seeded on a glass bottom petri dish and allowed to adhere overnight. Cells in 150 µl fresh tissue culture medium containing 1x EDTA-free protease inhibitors were placed on a stage of a confocal microscope (Leica SP5) equipped with a humidified chamber and equilibrated at 37°C (Life Imaging Services, Basel, Switzerland). A z-stack (0 time point) was recorded during 15 min of pre-incubation. The medium was then replaced by 150 µl of fresh tissue culture medium containing 30 nM CXCL12-Venus. Z-stacks were recorded every 2 min during 22 min. For each time point, planes corresponding to the center of the cells, excluding surface and bottom areas were collapsed and the fluorescence intensities above threshold of the YFP channels (CXCL12-venus) calculated with Metamorph software (Molecular Devices GmbH, Biberach an der Riss, Germany).

For FACS analysis of ligand internalization, 143B-*LacZ*-EV and 143B-*LacZ-*HA*-*X7 cells were detached with trypsin/ethylenediaminetetraacetic acid (EDTA) and resuspended in FACS buffer (1xPBS/0.05% EDTA/1% BSA). 200 µl of this suspension, with a density of 1 x10^6^ cells/ml, were used for individual experiments with the different Venus-fluorescent ligands. The cells were transferred to a 96 well plate, harvested by centrifugation and resuspended in FACS buffer containing the indicated Venus-fluorescent ligands. The cells were incubated at 37°C for 30 min and cells incubated on ice were used as a control. Uptake was stopped by centrifugation at 4°C for 2 min and ligand bound to CXCR7 at the cell surface was removed by a brief acidic wash with 150 mM NaCl, 50 mM glycine, pH 3. Internalized ligand was then quantified by FACS analysis.

### Agonist stimulated receptor internalization

143B-*LacZ*-HA-X7 cells were incubated with 0, 1, 10 or 100 nM CXCL12 and incubated at 37°C for 30 min. Cells were then subjected to an acidic wash (see above) to remove surface bound ligand. Receptors remaining localized at the cell surface were immunostained with CXCR7 antibodies [40] and Alexa Fluor 488-F(ab’) goat anti-mouse IgG (Invitrogen, Carlsbad, CA) and the cells subsequently sorted by FACS.

### Adhesion to endothelial cells

Human umbilical vein endothelial HUVECs cells were kindly provided by Dr. S.P. Hoerstrup, Swiss Center for Regenerative Medicine (SCRM), University Hospital, Zurich (License Number STV1-2005 approved by the local Ethics Committee) [41]. HUVECs were cultured in EGM-2 MV medium (Lonza, Basel, Switzerland) and seeded into 96-well plates (35’000/well) 24 h before the adhesion assay. 143B-*LacZ-*EV and 143B*-LacZ-*HA-X7 cells were labeled with Vybrant® Dil (Invitrogen, Carlsbad, CA) and reseeded in cell culture flasks. Adhesion to HUVEC was assessed in three independent experiments in triplicate wells for each condition and cell line. Briefly, labeled 143B-*LacZ-*EV and 143B*-LacZ-*HA-X7 cells grown in the cell culture flasks were detached and 10^4^ cells were added to untreated or IL-1β stimulated (1 nM, 6 h) HUVECs. 143B-*LacZ*-EV and 143B-*LacZ*-HA-X7 cells were then allowed to adhere at 37°C for 15 min in the absence of IL-1β. Non-adherent cells were removed by shaking the plates, and the wells were washed twice with PBS and fixed with 4% formalin at RT for 10 min. Adherent fluorescent cells were viewed with a Zeiss Observer. Z1 microscope equipped with appropriate fluorescence filter and counted with ImageJ software. The mean numbers of adhering cells of the individual cell lines at indicated conditions were calculated from the number of adhering cells counted in areas of 3.6 mm^2^ in respective wells (12% of total well area). The results of the three independent experiments are presented as per cent of adherent cells compared to seeded cells.

### Intratibial OS model in SCID mice

Female 6-8 week old immunosuppressed SCID mice were purchased from Charles River Laboratories (Sulzfeld, Germany) at least 7 days prior to experimental commencement. Housing conditions and experimental protocols were in accordance with institutional guidelines approved by the Ethics Committee of the Veterinary Department, Canton of Zurich, Switzerland (License Number 129/2009). The mice were housed in groups of 5 animals in individual ventilated 32 x 16 cm cages at room temperature. 143B-*LacZ*-EV and 143B-*LacZ-*HA*-*X7 cells were grown to subconfluency, detached with Trypsin/EDTA/PBS and resuspended in PBS/0.05% EDTA at a final density of 5x10^7^ cells/ml. Ten microliters of the cell suspension (5x10^5^ cells) were orthotopically injected into the medullar cavity of the left tibia of the mice. Tumor growth and osteolysis were examined by X-ray (Faxitron X-Ray Corporation, Lincolnshire, IL) once a week. The tumor volume was calculated from caliper rule measurements with the following formulas: leg volume = length x (width)^2^ x 0.5; tumor volume = leg volume on day X – leg volume leg on injection day 0; (X= 0,7,14,21 and 27 days). Organs were prepared as described previously [39]. The group of mice injected with 143B-*LacZ*-EV cells consisted of 6 animals and that injected with 143B-*LacZ*-HA-X7 cells of 7 animals. All mice in both groups developed intratibial primary tumors and metastases in the lung.

All mice were sacrificed on day 27. Lungs were prepared as described by Arlt et al. [38] and *LacZ* expressing metastatic tumor cells were visualized by X-Gal staining as described [42]. Briefly, lung tissue was refixed with 2% formaldehyde and 0.2% glutaraldehyde in PBS at room temperature for 1 h, washed 3-times with PBS and then stained in X-Gal staining solution at 37°C for at least 3 h. Metastases, defined as indigo-blue stained foci >0.1 mm in diameter on the surface of whole-mounts of lungs, were counted.

### Immunohistochemistry of mouse tissue

Heart, lung and legs were fixed with 4% paraformaldehyde and embedded in paraffin. Sections of 3-6µm were deparaffinized, rehydrated, counterstained in hematoxylin and washed with tap water. Subsequently, the sections were incubated with antibodies to the HA-tag of CXCR7 (Covance, Princeton, NJ), to CXCR7 (Proteintech, Chicago, IL), to CXCR4 (R&D Systems, Minneapolis, MN) to CXCL12 (K15C, kind gift of F. Arenzana-Seidedos, Paris). EnVision-method (DAKO, Glostrup, Denmark) was applied according to the manufacturer’s instructions and the peroxidase substrate AEC (Invitrogen, Carlsbad, CA) was used as a chromogen to visualize tissue staining.

### Statistical analyses

Results are presented as mean ± standard error of the mean (SEM). The data obtained from the experiments that assessed the adhesion to non-stimulated and IL-1β stimulated HUVEC cells were analyzed with one-way ANOVA (Bonferroni test). The values of primary tumor volume and the number of metastases were analyzed by the unpaired Student’s *t*-test or by Mann-Whitney-test with GraphPad Prism® 5.01 software.

## Supporting Information

Figure S1 **Immunostaining of paraffin sections confirmed overexpression of CXCR7 in the primary tumor of mice injected with 143B-*LacZ*-HA-X7 cells compared to the group injected with 143B-*LacZ*-EV whereas CXCR4 levels were comparable.** (A) Representative images of CXCR7 staining on primary tumor tissue from 143B-*LacZ*-EV (EV) and 143B-*LacZ*-HA-X7 (HA-X7) cell injected mice. (B) Representative images of CXCR4 staining on primary tumor tissue from 143B-*LacZ*-EV (EV) and 143B-*LacZ*-HA-X7 (HA-X7) cell injected mice. In panels A and B negative controls show images of sections of primary tumors stained with the secondary antibody alone. Size bars: 50 µm.
(TIF)

Movie S1 **143B-*LacZ*-HA-X7 cells demonstrate efficient uptake of CXCL12 –Venus compared to** **143B-*LacZ*-EV cells due to the scavenging activity of CXCR7** (S1). Video showing time-dependent uptake of CXCL12 –Venus by 143B-*LacZ*-HA-X7 cells (S2). Video showing a very weak time-dependent uptake of CXCL12 –Venus by 143B-*LacZ*-EV cells.
(AVI)

Movie S2 **Video showing time-dependent uptake of CXCL12 –Venus by 143B-*LacZ*-HA-X7 cells.** (S2) Video showing a very weak time-dependent uptake of CXCL12 –Venus by 143B-*LacZ*-EV cells.
(AVI)
