# Supplementary figures and images for: Expression of the Chemokine Receptor CXCR7 in CXCR4-Expressing Human 143B Osteosarcoma Cells Enhances Lung Metastasis of Intratibial Xenografts in SCID Mice
Source: PLoS One. 2013 Sep 10;8(9):e74045. doi: 10.1371/journal.pone.0074045 (PMC3769403; doi:10.1371/journal.pone.0074045)

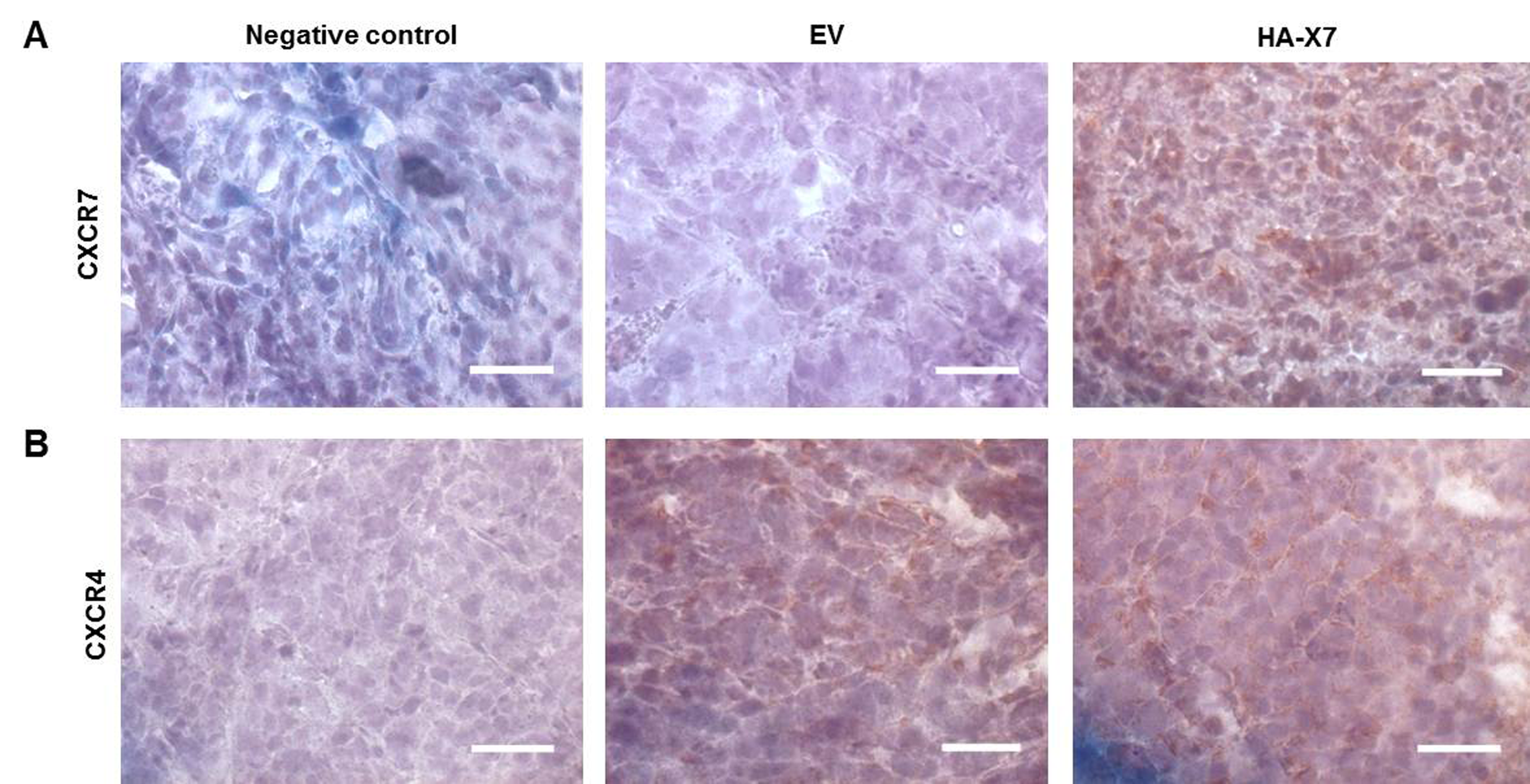

Supplement: Figure S1 — Immunostaining of paraffin sections confirmed overexpression of CXCR7 in the primary tumor of mice injected with 143B-LacZ-HA-X7 cells compared to the group injected with 143B-LacZ-EV whereas CXCR4 levels were comparable. (A) Representative images of CXCR7 staining on primary tumor tissue from 143B-LacZ-EV (EV) and 143B-LacZ-HA-X7 (HA-X7) cell injected mice. (B) Representative images of CXCR4 staining on primary tumor tissue from 143B-LacZ-EV (EV) and 143B-LacZ-HA-X7 (HA-X7) cell injected mice. In panels A and B negative controls show images of sections of primary tumors stained with the secondary antibody alone. Size bars: 50 µm. (TIF) [file pone.0074045.s001.tif]
